# COVID-19 Vaccine Acceptability Among Healthcare Facility Workers in Sierra Leone, the Democratic Republic of Congo and Uganda: A Multi-Centre Cross-Sectional Survey

**DOI:** 10.3389/ijph.2022.1605113

**Published:** 2022-09-23

**Authors:** Hilary S. Whitworth, Jonathan Kitonsa, Kambale Kasonia, Daniel Tindanbil, Paddy Kafeero, Joseph Bangura, Yusupha Nije, Darius Tetsa Teta, Brian Greenwood, Hugo Kavunga-Membo, Bailah Leigh, Eugene Ruzagira, Katherine E. Gallagher, Deborah Watson-Jones

**Affiliations:** ^1^ London School of Hygiene and Tropical Medicine, University of London, London, United Kingdom; ^2^ Medical Research Council (MRC)/Uganda Virus Research Institute (UVRI) and London School of Hygiene and Tropical Medicine (LSHTM) Uganda Research Unit, Entebbe, Uganda; ^3^ Institut National de Recherche Biomédicale (République démocratique du Congo), Kinshasa, Democratic Republic of Congo; ^4^ College of Medicine and Allied Health Sciences, University of Sierra Leone, Freetown, Sierra Leone; ^5^ Ministry of Health and Sanitation (Sierra Leone), Freetown, Sierra Leone; ^6^ KEMRI Wellcome Trust Research Programme, Kilifi, Kenya; ^7^ Mwanza Intervention Trials Unit, Mwanza, Tanzania

**Keywords:** healthcare workers, sub-Saharan Africa, COVID-19 pandemic, COVID-19 vaccines, vaccine uptake, vaccine acceptability

## Abstract

**Objectives:** This cross-sectional survey explored COVID-19 vaccine acceptability among public healthcare facility workers in Kambia (Sierra Leone), Goma (Democratic Republic of Congo) and Masaka (Uganda).

**Methods:** Questionnaire-based interviews conducted between April–October 2021 explored participants’ knowledge and perceptions of, and attitudes towards, the COVID-19 pandemic and COVID-19 vaccines, as well as COVID-19 vaccine acceptability (defined as uptake of ≥1 dose or intent to get vaccinated).

**Results:** Whilst most (*n* = 444; 81.8%) of the 543 participants had one or more concerns about COVID-19 vaccines, 487 (89.7%) nonetheless perceived that they were important for pandemic control. Most participants from Kambia or Masaka either were vaccinated (*n* = 137/355; 38.6%) or intended to get vaccinated (*n* = 211/355; 59.4%) against COVID-19. In Goma, all 188 participants were unvaccinated; only 81 (43.1%) participants intended to get vaccinated, and this was associated with positive perceptions about COVID-19 vaccines. In Goma, the most common reasons for not wanting a COVID-19 vaccine were concerns that the vaccines were new (*n* = 75/107; 70.1%) and fear of side effects (*n* = 74/107; 69.2%).

**Conclusion:** Reported COVID-19 vaccine acceptability was high among healthcare facility workers in Kambia and Masaka. The lower vaccine acceptability in Goma may highlight the importance of social mobilisation and accurate, accessible information that addresses specific concerns.

## Introduction

The development, licensing and implementation of prophylactic COVID-19 vaccines was an immense global accomplishment. Up to May 2022, over 66% of the global population had received at least 1 COVID-19 vaccine dose, but vaccine uptake varied considerably across regions and countries, ranging from just 0.1% in Burundi to 99% in the United Arab Emirates [1, 2]. Whilst funding constraints and inequitable vaccine access have undoubtedly resulted in slower vaccine distribution in many low and middle income countries (LMIC) [3, 4], vaccine hesitancy has presented a separate challenge in both high income countries (HIC) and LMIC [5, 6]. Some countries in Sub-Saharan Africa (SSA) have come under the spotlight with reported widespread reluctance to be vaccinated that has led to COVID-19 vaccines expiring and being destroyed [7, 8].

Until recently, the scientific community’s assumption was that vaccine hesitancy arises from misinformation and lack of access to correct information; however, an important role of institutional and social mistrust is increasingly recognised [6, 9], which has been particularly evident during the COVID-19 pandemic [10–12]. Healthcare workers are often trusted figureheads within communities and thus have an integral role in promoting vaccine uptake [6, 13, 14]. Furthermore, healthcare workers are widely considered as a priority group for COVID-19 vaccination due to their high risk of exposure to SARS-CoV-2, their contact with vulnerable individuals, and the burden on healthcare services if they are off work due to sickness.

Few studies have evaluated COVID-19 vaccine acceptability among healthcare workers in SSA and the sparse data available have demonstrated variable acceptability in this group, though some of the studies were conducted before COVID-19 vaccines were actually available to participants [15, 16]. This was a multi-centre cross-sectional survey to evaluate COVID-19 vaccine acceptability (inclusive of vaccine uptake or intent to get vaccinated) among public healthcare facility workers in three countries spanning West, Central and East Africa—Sierra Leone, Democratic Republic of Congo (DRC) and Uganda—in the months following vaccine introduction in these countries. The study explored participants’ knowledge and perceptions of, and attitudes towards, COVID-19 vaccines in order to better understand drivers of vaccine acceptability, and evaluated factors specifically associated with COVID-19 vaccine uptake or intent to get vaccinated.

## Methods

### Study Design

This was a cross-sectional survey nested within the serosurvey component of a study assessing the impact of COVID-19 on primary health care service provision and utilisation in Kambia (Sierra Leone), Goma (DRC) and Masaka (Uganda): the “COVID-19 HWI study.” The COVID-19 HWI study has been described elsewhere [17]. In the serosurvey component, repeated SARS-CoV-2 serology was conducted among staff with patient contact roles at participating healthcare facilities to estimate their exposure to SARS-CoV-2, the incidence of infection during the study period, and the rate of antibody waning following infection. The vaccine acceptability survey described in this paper was conducted among healthcare facility workers when they were participating in the second or third serosurvey.

### Study Setting

Kambia district, located in the northern province of Sierra Leone, covers an area of ∼3,100 km^2^ and has ∼345,000 residents and 69 public health facilities (1 hospital, 15 community health centres, 16 community health posts, and 37 maternal child health posts). Goma, the capital of the North Kivu province of the DRC, covers an area of ∼76 km^2^ and has ∼707,000 residents and 39 public health facilities (13 tertiary care hospitals and 26 health centres). Masaka, located in southern Uganda, covers an area of ∼1,603 km^2^ and has ∼336,000 residents and 36 health facilities (2 hospitals, 25 public health centres and 9 private not-for-profit health centres). Twenty-nine public health facilities in Kambia were selected for participation in the COVID-19 HWI study through stratified random sampling to ensure proportional representation of health posts and health facilities [17]. All 25 public health centres in Masaka and 21 accessible health centres in Goma were selected for participation.

Details of the COVID-19 pandemic situation and COVID-19 vaccine introductions in the 3 study countries are shown in Table 1.

**TABLE 1:** Details on the COVID-19 pandemic situation and COVID-19 vaccine roll-out in Sierra Leone, Democratic Republic of Congo and Uganda at the time when the survey was conducted and up to May/June 2022. (COVID-19 vaccination in health workers; Sierra Leone, Democratic Republic of Congo and Uganda; 2021).

	Sierra Leone	DRC	Uganda
Population size^a^	8.0 million	89.6 million	45.7 million
First identified COVID-19 case	31st March 2020	10th March 2020	22nd March 2020
COVID-19 vaccines available in country	March 2021	April 2021^g^	March 2021
Vaccine acceptability survey conducted	19 April–24 June 2021	23 June-27July 2021	7 September–8 October 2021
Covid-19 situation and vaccine roll-out in the country at survey commencement			
Number of infection waves^b^	2	3 (mid 3rd wave)	2
Confirmed COVID-19 cases^b^			
Number of cases	4,038	38,553	120,714
Cases per million population	496	417	2,562
Confirmed COVID-19 deaths^b^			
Number of deaths	79	891	3,061
Deaths per million population	10	10	65
Vaccination progress^c^			
% of population with ≥1 vaccine dose	0.52%	0.08%	2.19%
% of population fully vaccinated^f^	0.02%	<0.01%	0.78%
Covid-19 situation and vaccine roll-out in the country up to May/June 2022			
Number of infection waves^d^	4	4^h^	3^h^
Confirmed COVID-19 cases^d^			
Number of cases	7,683	89,189	165,927
Cases per million population	944	965	3,521
Confirmed COVID-19 deaths^d^			
Number of deaths	125	1,338	3,602
Deaths per million population	15	14	76
Vaccination progress^e^			
% of population with ≥1 vaccine dose	27.0%	2.1%	33.4%
% of population fully vaccinated^f^	18.1%	1.3%	22.6%

DRC, democratic Republic of Congo.

a Based on 2020 data from the World Bank Group [37].

b Based on data from Our World in Data, correct at 19th April 2021 for Sierra Leone, 23rd June 2021 for the DRC and 7th September 2021 for Uganda [38].

c Based on data from Our World in Data, correct at 15th April 2021 for Sierra Leone, 20th June 2021 for the DRC, and 5th September 2021 for Uganda [1].

d Based on data from Our World in Data, correct at 9th June 2022 for all three countries [38].

e Based on data from Our World in Data, correct at 22nd May 2022 for all three countries [1].

f Whereby fully vaccinated refers to a complete initial protocol.

g The COVID-19 vaccine launch was initially scheduled for 15th March 2021, shortly after a shipment of 1.7 million doses of the AstraZeneca vaccine arrived in the country. However, the launch was paused for one month in the DRC following the temporary suspension of the AstraZeneca vaccine across multiple European countries the same month (from 7th March 2021) [24]. The DRC’s vaccination campaign started in April 2021, with the first phase of vaccinations targeting Kinshasa, North Kivu (where Goma is located), Central Kongo and Haut-Katanga—the provinces most affected by the pandemic.

h Case numbers of COVID-19 were starting to increase in the DRC and Uganda at the time of writing, possibly signalling the start of the next infection wave in these countries.

### Vaccine Survey Participants

Selection of participants for the COVID-19 HWI serosurvey has been described previously [17]. Healthcare facility workers aged ≥18 years who were enrolled in the COVID-19 HWI serosurvey and available at the time that the vaccine acceptability survey was conducted were eligible for inclusion. Potential participants were provided with an information sheet about the study, and written informed consent was obtained from all individual participants included in the study.

### Data Collection and Study Variables

Data were collected through structured, face-to-face questionnaire-based interviews conducted by trained study staff members. The questionnaire, which was developed by the study team specifically for this survey, was pre-programmed into computer tablets. Participants were asked about their knowledge and perceptions of, and attitudes towards, the COVID-19 pandemic and COVID-19 vaccines, their COVID-19 vaccination status and, if unvaccinated, whether they would accept COVID-19 vaccination if it were offered to them. The interviewer read each question to the participant and pre-coded answers were recorded in the electronic case report form.

### Statistical Analysis

Data were analysed using STATA version 16.0. Data were summarised descriptively and tabulated, stratified by country. Primary analyses were conducted among all healthcare facility roles combined. Secondary analyses were stratified by role, considering level and proximity of patient contact as follows [1]: doctors, clinical officers, nurses and midwives—highest contact [2]; clinical support staff (such as health attendants, antenatal care workers and community health workers)—medium contact; and [3] pharmacy and laboratory staff and non-clinical support staff (such as ambulance drivers, porters and receptionists)—lowest contact.

Multivariable logistic regression was used to examine factors associated with COVID-19 vaccine uptake or intent to get vaccinated. Regression analyses first evaluated associations with age, sex, education level and role in the healthcare facility. Second, adjusted for these variables, analyses examined whether vaccine uptake or intent to get vaccinated was associated with specific perceptions on the impact of the COVID-19 pandemic and the COVID-19 response in the country, as well as understanding and views of COVID-19 vaccines.

### Ethics

The study was reviewed and approved by the London School of Hygiene and Tropical Medicine Research Ethics Committee (Ref: 22726), the Uganda Virus Research Institute Research Ethics Committee (Ref: GC/127/821), the Uganda National Council for Science and Technology (Ref: H1430ES), the Comité National d’Ethique de la Santé, and the Sierra Leone Ethics and Scientific Review committee. All study procedures were performed in accordance with the ethical standards of the institutional and national research committees, and with the 1964 Helsinki declaration and its later amendments or comparable ethical standards.

## Results

The vaccine acceptability survey was conducted from 19th April to 24th June 2021 in Kambia (1–3 months after COVID-19 vaccine introduction in Sierra Leone), 23rd June to 27th July 2021 in Goma (2-3 months after vaccine introduction in the DRC), and 7th September to 8th October 2021 in Masaka (6–7 months after vaccine introduction in Uganda) (Table 1). In total, 543 healthcare facility staff participated, 124 (22.8%) from Kambia, 188 (34.6%) from Goma and 231 (42.5%) from Masaka. Brief demographics and characteristics of the interviewees are shown in Table 2.

**TABLE 2:** Participant demographics and characteristics. (COVID-19 vaccination in health workers; Sierra Leone, Democratic Republic of Congo and Uganda; 2021).

	Kambia	Goma	Masaka	Total
	N = 124	N = 188	N = 231	N = 543
Age in years, median (range)^a^	38 (20-68)	38 (18-75)	35 (18-74)	37 (18-75)
Sex				
Male	44 (35.5)	82 (43.6)	73 (31.6)	199 (36.7)
Female	80 (64.5)	106 (56.4)	158 (68.4)	344 (63.4)
Role in HCF ^b^				
Doctor or clinical officer	0	4 (2.1)	12 (5.2)	16 (3.0)
Nurse or midwife^c^	29 (23.4)	152 (80.9)	120 (52.0)	301 (55.4)
Clinical support staff^d^	71 (57.3)	13 (6.9)	48 (20.8)	132 (24.3)
Laboratory and pharmacy staff^e^	12 (9.7)	18 (9.6)	30 (13.0)	60 (11.1)
Non-clinical support staff^f^	12 (9.7)	1 (0.5)	21 (9.1)	34 (6.3)
Highest level of schooling				
None^g^	7 (5.7)	0	1 (0.4)	8 (1.5)
Complete primary	1 (0.8)	1 (0.5)	7 (3.0)	9 (1.7)
Incomplete secondary	31 (25.0)	15 (8.0)	20 (8.7)	66 (12.2)
Complete secondary and above	85 (68.5)	172 (91.5)	203 (87.9)	460 (84.7)
Believes that vaccines are important public health interventions				
Yes	124 (100.0)	186 (98.9)	229 (99.1)	539 (99.3)
No	0	2 (1.1)	2 (0.9)	4 (0.7)

N, number; HCF, healthcare facility.

a Data are missing for one participant from Masaka.

b All roles include trainees.

c Includes registered, enrolled or assistant nurses and midwives, and community health nurses.

d Includes health attendants and assistants, maternal and child health aids, antenatal care workers, community health workers, counsellors, peer/health educators, nutritionists, physiotherapists, psychologists, social workers and triage/screening staff.

e Includes laboratory technicians and assistants, and pharmacists and pharmacy attendants.

f Includes community linkage personnel, ambulance drivers, data clerks, health information assistants, health inspectors, porters, receptionists, retention officers, cleaners and other support staff.

g In Kambia, 3 participants reporting no education were traditional birth attendants, 2 were laboratory assistants, 1 was a porter and 1 had a role in patient triage. In Masaka, the 1 participant who reported no education was a cleaner.

Overall, 99.3% of participants agreed that vaccines in general (i.e., not only COVID-19 vaccines) are important public health interventions. Most agreed that vaccines can prevent infections (*n* = 433/539; 80.3%) and severe illness or death from some diseases (*n* = 490/539; 90.9%), and that they can reduce the burden on healthcare services (414/539; 76.8%) and healthcare costs (389/539; 72.2%). The few participants (2 registered nurses in Goma and 1 counsellor and a health information assistant from Masaka) who did not agree that vaccines, in general, are important public health interventions had concerns over vaccine safety (n = 3) and efficacy (*n* = 2) and/or considered vaccine manufacturers to be untrustworthy (*n* = 3).

### Knowledge and Perceptions About the COVID-19 Pandemic and COVID-19 Vaccines

All participants in Kambia, 185 (98.4%) from Goma and 225 (97.4%) from Masaka agreed that COVID-19 was an important public health problem in their country (Table 3). Results were similar when stratifying by role in the healthcare facility (Supplementary Tables S1–S3). Most participants perceived that many healthcare workers were getting sick (337/543; 62.1%), and that many people generally were becoming unwell (*n* = 455/543; 83.8%) and dying (*n* = 365/545; 67.2%), from COVID-19 in their country. However, 2 registered nurses from Goma and a training nurse from Masaka believed that COVID-19 was not a real disease (*n* = 2), or that there was no SARS-CoV-2 or COVID-19 in their country (*n* = 2).

**TABLE 3:** Knowledge and perceptions of the COVID-19 pandemic and COVID-19 vaccines.^a^ (COVID-19 vaccination in health workers; Sierra Leone, Democratic Republic of Congo and Uganda; 2021).

	Kambia	Goma	Masaka	Total
	N = 124	N = 188	N = 231	N = 543
Perceptions of COVID-19 as a public health problem in this country				
COVID-19 is an important public health problem	124 (100.0)	185 (98.4)	225 (97.4)	534 (98.3)
Many people are getting sick from COVID-19	117 (94.4)	161 (85.6)	177 (76.6)	455 (83.8)
Many people are dying from COVID-19	75 (60.5)	143 (76.1)	147 (63.6)	365 (67.2)
Many HCW are getting sick from COVID-19	110 (88.7)	81 (43.1)	146 (63.2)	337 (62.1)
Many HCW are dying from COVID-19	75 (60.5)	53 (28.2)	103 (44.6)	231 (42.5)
HCF are overwhelmed with COVID-19 cases	57 (46.0)	26 (13.8)	107 (46.3)	190 (35.0)
Perceptions of COVID-19 impact in this country				
Healthcare services are suffering	65 (52.4)	39 (20.7)	184 (79.7)	288 (53.0)
Other diseases are more important	78 (62.9)	81 (43.1)	45 (19.5)	204 (37.6)
COVID-19 response is causing neglect of other diseases	54 (43.6)	32 (17.0)	135 (58.4)	221 (40.7)
COVID-19 response is detrimental to the economy	119 (96.0)	79 (42.0)	199 (86.2)	397 (73.1)
COVID-19 response is detrimental to education	65 (52.4)	140 (74.5)	216 (93.5)	421 (77.5)
Knowledge and perceptions of COVID-19 vaccines				
They are under development/being evaluated	117 (94.4)	165 (87.8)	116 (50.2)	398 (73.3)
They are licensed and used in some countries	122 (98.4)	120 (63.8)	153 (66.2)	395 (72.7)
They are available in this country	86 (69.4)	144 (76.6)	176 (76.2)	406 (74.8)
They protect against virus that causes COVID-19	116 (93.6)	42 (22.3)	120 (52.0)	278 (51.2)
They stop people getting very sick from COVID-19	116 (93.6)	42 (22.3)	123 (53.3)	281 (51.8)
They are important for control of the pandemic	124 (100.0)	141 (75.0)	222 (96.1)	487 (89.7)
Advantages of COVID-19 vaccines				
They can/may protect healthcare workers	123 (99.2)	147 (78.2)	193 (83.6)	463 (85.3)
They can be given to lots of people quickly	117 (94.4)	43 (22.9)	94 (40.7)	254 (46.8)
They may allow travel/movement/socializing	123 (99.2)	128 (68.1)	159 (68.8)	410 (75.5)
They may encourage visitors from other countries	123 (99.2)	118 (62.8)	147 (63.6)	388 (71.5)
They may allow the economy to recover	92 (74.2)	89 (47.3)	138 (59.7)	319 (58.8)
Disadvantages of COVID-19 vaccines				
Vaccine rollout is a burden on healthcare services	111 (89.5)	44 (23.4)	62 (26.8)	217 (40.0)
They are new and experimental	113 (91.1)	166 (88.3)	114 (49.4)	393 (72.4)
They may cause side effects/harm	124 (100.0)	129 (68.6)	149 (64.5)	402 (74.0)
Multidose vaccine schedules are difficult to deliver	113 (91.1)	24 (12.8)	84 (36.4)	221 (40.7)
They are expensive; big cost to the country	112 (90.3)	50 (26.6)	125 (54.1)	287 (52.9)
Concerns over COVID-19 vaccines				
They may cause infertility	95 (76.6)	39 (20.8)	33 (14.3)	167 (30.8)
They may cause SARS-CoV-2 infection/COVID-19	91 (73.4)	107 (56.9)	52 (22.5)	250 (46.0)
They may cause symptoms like COVID-19	93 (75.0)	132 (70.2)	111 (48.1)	336 (61.9)
They may affect pregnancies/foetuses	96 (77.4)	99 (52.7)	88 (38.1)	283 (52.1)
They may cause anaphylaxis	96 (77.4)	120 (63.8)	68 (29.4)	284 (52.3)
They may cause other harm	91 (73.4)	105 (55.9)	41 (18.2)	238 (43.8)
They may not work	89 (71.8)	42 (22.3)	32 (13.9)	163 (30.0)

N, number; HCF, healthcare facility; HCW, healthcare workers.

a For all variables, participants could provide more than one answer so summed percentages are not equal to 100%.

Views on the impact of the COVID-19 pandemic varied considerably across the 3 study sites (Table 3). For example, 184 (79.7%) participants in Masaka believed that healthcare services in their country were suffering because of the pandemic, compared with just 39 (20.7%) from Goma. Most (*n* = 119; 96.0%) participants from Kambia considered that the COVID-19 response was detrimental to their country’s economy, compared to just 79 (42.0%) from Goma.

Most participants (89.7% overall; 86.4% of clinicians, nurses and midwives) considered that COVID-19 vaccines were important to control the pandemic; this ranged from 75.0% of participants in Goma to 100.0% in Kambia (Table 3; Supplementary Tables S1–S3). Among the participants who did not agree that COVID-19 vaccines were important for control of the pandemic, the most commonly cited reasons were that there were other priorities that were more important for the country (*n* = 15), that COVID-19 could be controlled with other measures (*n* = 15) and that COVID-19 vaccines might not work (*n* = 15). Overall, 58 (46.8%) participants from Kambia, 21 (11.2%) from Goma and 154 (66.7%) from Masaka believed that COVID-19 vaccination should be compulsory in their country.

Whilst the results above indicate that overall support for COVID-19 vaccines was high (albeit lower in Goma compared to the other study sites), 393 (72.4%) participants (ranging from 49.4% in Masaka to 91.1% in Kambia) had concerns that the vaccines were new and experimental (Table 3). Findings were similar when restricted to clinicians, nurses and midwives (Supplementary Tables S1–S3). Overall, 250 (46.0%) participants had concerns that COVID-19 vaccines could cause SARS-CoV-2 infection or COVID-19, 167 (30.8%) had concerns that they could cause infertility and 283 (52.1%) had concerns that they could be harmful in pregnancy; all of these concerns were more common in Kambia than in Goma or Masaka (Table 3). Findings were similar when restricted to clinicians, nurses and midwives (Supplementary Tables S1–S3). Only 174 (54.9%) participants from this restricted group considered that COVID-19 vaccines could cause anaphylaxis, despite this being a known risk associated with vaccines.

### COVID-19 Vaccine Uptake and Acceptability

**FIGURE 1:**
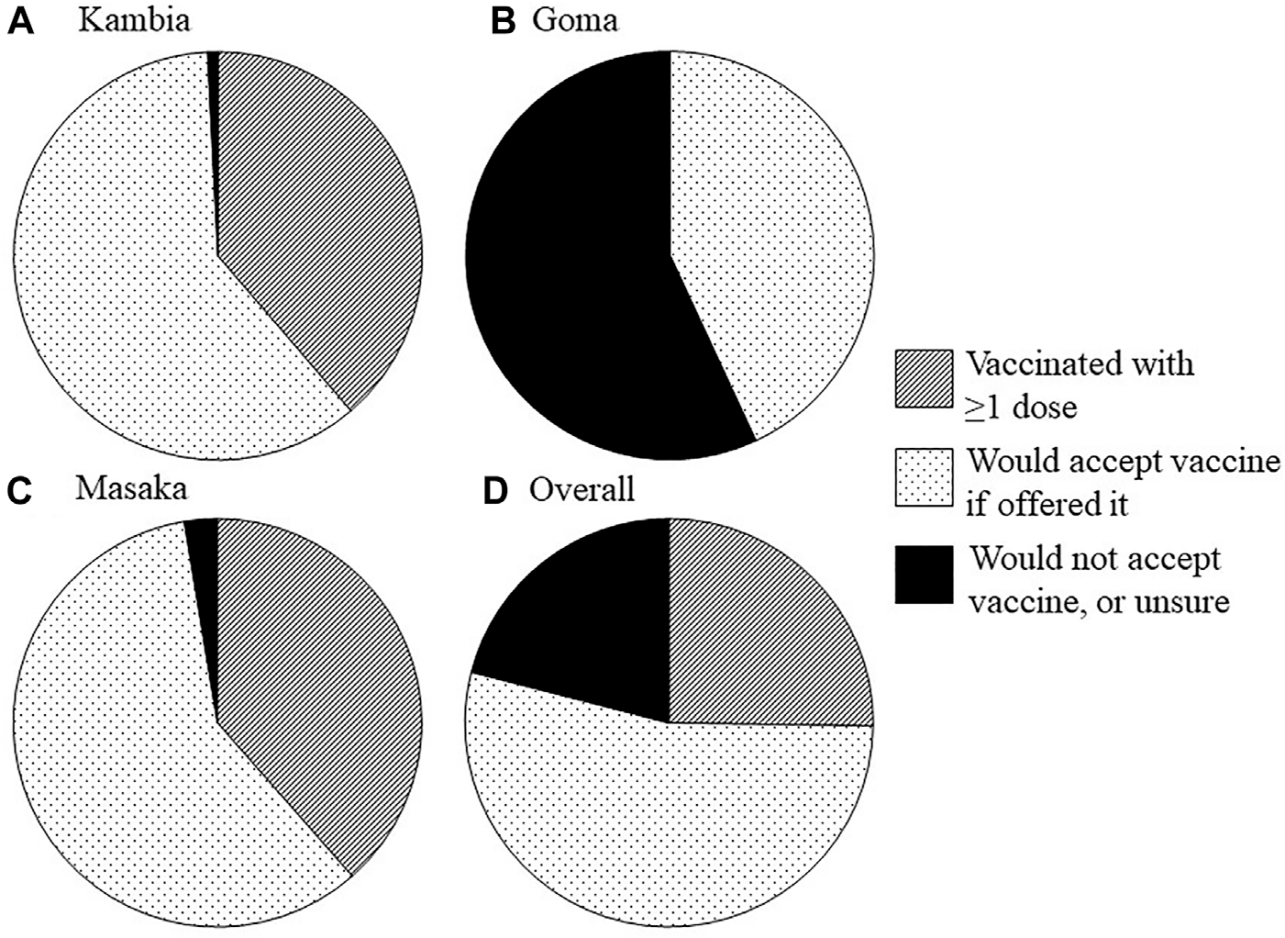
COVID-19 vaccine uptake, intent to get vaccinated and vaccine hesitancy/declining vaccination among healthcare facility workers. Pie charts show, by study site [**(A)** Kambia, **(B)**: Goma, **(C)**: Masaka, and **(D)**: overall], the proportion of participants who had already received at least one dose of the vaccine by the time of the survey, who would accept the vaccine if offered it and who would not accept the vaccine or did not know if they would accept the vaccine. (COVID-19 vaccination in health workers; Sierra Leone, Democratic Republic of Congo and Uganda; 2021).

At the time of the survey, 48 (38.7%) participants from Kambia and 89 (38.5%) from Masaka had received ≥1 COVID-19 vaccine dose (Table 4; Figure 1). In Kambia, vaccine uptake (≥1 dose) was highest among clinicians, nurses and midwives (*n* = 17/29; 58.6%), followed by clinical support workers (*n* = 25/71; 35.2%), followed by other workers (*n* = 6/24; 25.0%) (37.9%) (Chi^2^ = 7.1, *p* = 0.029; Supplementary Table S1). There was no difference in uptake between different roles in Masaka (Supplementary Table S3). Most unvaccinated participants from Kambia (*n* = 75/76; 98.7%) and Masaka (*n* = 136/142; 95.8%) said that they would accept a COVID-19 vaccine if offered it, meaning that reported willingness to accept the vaccine—including either uptake of ≥1 dose by the time of the survey or intent to get vaccinated—was very high (99.2% in Kambia; 97.4% in Masaka). In Goma, no study participant was vaccinated against COVID-19 at the time of the survey (despite the vaccine being available to healthcare facility workers at that time) and only 81 (43.1%) participants (and 43.6% of clinicians, nurses and midwives) said that they would accept the vaccine if offered it.

**TABLE 4:** COVID-19 vaccine uptake and acceptability. (COVID-19 vaccination in health workers; Sierra Leone, Democratic Republic of Congo and Uganda; 2021).

	Kambia	Goma	Masaka	Total
	N = 124	N = 188	N = 231	N = 543
COVID-19 vaccination status				
Fully vaccinated^a^ ^,^ ^b^	9 (7.3)	0	34 (14.7)	43 (7.9)
Partially vaccinated^a^ ^,^ ^b^	39 (31.5)	0	55 (23.8)	94 (17.3)
Unvaccinated—would accept vaccine	75 (60.5)	81 (43.1)	136 (58.9)	294 (53.9)
Unvaccinated—would not accept vaccine	1 (0.8)	91 (48.4)	4 (1.7)	96 (17.7)
Unvaccinated—unsure if would accept vaccine	0	16 (8.5)	2 (0.9)	18 (3.3)
Reasons for accepting or being willing to accept the vaccine^c^ ^,^ ^d^				
To protect own health	123 (99.2)	81 (43.1)	214 (92.6)	418 (77.0)
To keep working	122 (98.4)	49 (26.1)	100 (43.3)	271 (49.9)
To avoid infecting other people	123 (99.2)	79 (42.0)	161 (69.7)	363 (66.9)
To protect family	122 (98.4)	76 (40.4)	172 (74.5)	370 (68.1)
Reasons for unwillingness to accept vaccine^c^ ^,^ ^d^				
Not at risk of catching SARS-CoV-2	1 (0.8)	18 (9.6)	2 (0.9)	21 (3.9)
Not at risk of becoming very ill or dying from COVID-19	1 (0.8)	18 (9.6)	0	19 (3.5)
Already had COVID-19	0	2 (1.1)	1 (0.4)	3 (0.6)
Frightened of vaccine side effects	1 (0.8)	74 (39.4)	5 (2.2)	80 (14.7)
COVID-19 vaccines are new and experimental	0	75 (39.9)	6 (2.6)	81 (14.9)
COVID-19 vaccines don’t work	0	30 (16.0)	2 (0.9)	32 (5.9)
Currently pregnant/breastfeeding	0	3 (1.6)	0	3 (0.6)
Factors that would influence decision on whether to get vaccinated^c^ ^,^ ^e^				
Which country the vaccine was developed in	100 (80.7)	105 (55.9)	129 (55.8)	334 (61.5)
Which company made the vaccine	92 (74.2)	88 (46.8)	100 (43.3)	280 (51.6)
Where the clinical trials were conducted	98 (79.0)	138 (73.4)	105 (45.5)	341 (62.8)
Whether clinical trials were conducted here	92 (74.2)	118 (62.8)	87 (37.7)	297 (54.7)
How many people were vaccinated before	91 (73.4)	127 (67.6)	73 (31.6)	291 (53.6)
How long the vaccine had been trialled for	110 (88.7)	134 (71.3)	111 (48.1)	355 (65.4)
What type of vaccine it is	85 (68.6)	73 (38.8)	109 (47.2)	267 (49.2)
Which other countries are giving the vaccine	55 (44.4)	90 (47.9)	72 (31.2)	217 (40.0)

N, number.

a In Kambia, vaccination status was confirmed for all 48 vaccinated participants using vaccination cards. In Masaka, vaccination status was confirmed for 13 vaccinated participants using vaccination cards, and 76 participants reported that they were vaccinated verbally.

b 79.6% of participants who received at least one vaccine dose were given the AstraZeneca vaccine (50.0% in Kambia; 95.5% in Masaka). In Kambia, all other vaccinated participants received the Sinopharm vaccine; in Masaka, all other vaccinated participants received the Sinovac vaccine.

c Participants could provide more than one answer so summed percentages are not equal to 100%.

d Participants provided either reasons for or against vaccination as appropriate to their answer for the vaccination status variable above, but proportions were calculated as percentages of the whole study population (by country and across all countries).

e Results are shown for all participants, including participants who had already received one or more COVID-19 vaccine doses.

Reasons for accepting or not accepting the COVID-19 vaccine (with “acceptance” referring to either vaccine uptake or intent to get vaccinated) are shown in Table 4. Among the 96 participants from the 3 study sites who reported that they would not accept the vaccine, the most common reasons were fear of side effects (*n* = 80; 83.3%) and concerns that the vaccines were new and experimental (*n* = 81; 84.4%). Approximately one fifth of participants who would not accept the vaccine believed that they were not at risk of catching SARS-CoV-2 (*n* = 21; 21.9%) or becoming seriously ill or dying from COVID-19 (*n* = 19; 19.8%), and almost a third believed that the vaccines did not work (*n* = 32; 33.3%).

When asked, hypothetically, what factors might influence participants’ decisions on whether or not to receive a COVID-19 vaccine, the most commonly selected factors included which country the vaccine was developed in (*n* = 334; 61.5%) and where and for how long the clinical vaccine trials to evaluate it were conducted (*n* = 341; 62.8% and *n* = 355; 65.4%, respectively), although results varied slightly across the 3 study sites (Table 4). Among the 114 unvaccinated participants who said that they would not accept a COVID-19 vaccine or were unsure if they would accept one, most said that, hypothetically, their decision on whether to get vaccinated might be influenced by where and for how long the clinical vaccine trials were conducted (*n* = 78; 68.4% and *n* = 74; 64.9%, respectively), and how many people had been vaccinated before them (*n* = 69; 60.5%).

In Kambia, theoretical willingness to be vaccinated against COVID-19 was high for vaccines developed in the United States (*n* = 119; 96.0%), the United Kingdom (*n* = 122; 98.4%), Europe (*n* = 112; 90.3%) or Russia (*n* = 113; 91.1%), but was lower for vaccines developed in China (*n* = 80; 64.5%) (Supplementary Figure S1). A similar pattern was observed in Goma (the United States: *n* = 95; 50.5%, United Kingdom: *n* = 92; 48.9%, Europe: *n* = 86; 45.7%, China: *n* = 57; 30.3%, Russia: *n* = 69; 36.7%). In Masaka, willingness to be vaccinated against COVID-19 was highest for vaccines developed in the United States (*n* = 192; 83.1%) and the United Kingdom (*n* = 185; 80.1%), and lower for those developed in Europe (*n* = 114; 49.4%), China (*n* = 97; 42.0%) or Russia (*n* = 100; 43.3%).

### Factors Associated With Intent to Get Vaccinated in Goma

Since almost all participants in Kambia and Masaka had either received ≥1 COVID-19 vaccine dose or reported their intention to receive the vaccine, analyses of factors associated with willingness to receive a COVID-19 vaccine were limited to participants from Goma (Table 5). Adjusted for age, sex and type of role within the healthcare facility, participants with lower education level were more likely to report intent to receive the COVID-19 vaccine than those with higher education level (OR 3.17, 95%CI 1.01–9.96; *p* = 0.040) (Table 5). Adjusted for age, sex, education and type of role within the healthcare facility, intention to get vaccinated was associated with participant perceptions that the COVID-19 pandemic, or the country’s response to the pandemic, was having a negative or detrimental affect on healthcare services (OR 5.69, 95%CI 2.45–13.21; *p* < 0.001), that the COVID-19 response was resulting in the neglect of other diseases (OR 8.61, 95%CI 3.24–22.89; *p* < 0.001), and that the COVID-19 response was detrimental to the economy (OR 2.24, 95%CI 1.21–4.17; *p* = 0.010). Intent to be vaccinated was also associated with participants’ perceptions/knowledge that COVID-19 vaccines protect against SARS-CoV-2 infection or can stop people from getting very ill from COVID-19 (for each, OR 3.69, 95%CI 1.73–7.87; *p* < 0.001), and that the vaccines are important for control of the pandemic (OR 18.19, 95%CI 5.23–63.30; *p* < 0.001).

**TABLE 5:** Factors associated with intent to get vaccinated against COVID-19 in Goma. (COVID-19 vaccination in health workers; Sierra Leone, Democratic Republic of Congo and Uganda; 2021).

Variable	Category	N intent to get vaccinated/Total (%)	Crude OR (95% CI)	LRT *p*-value	Adjusted OR (95% CI)	LRT *p*-value
Age in years^a^	≤35	28/79 (35.4)	1.0	0.195	1.0	0.244
	36–50	39/80 (48.8)	1.73 (0.92–3.27)		1.75 (0.91–3.39)	
	>50	14/29 (48.3)	1.70 (0.72–4.03)		1.35 (0.54–3.37)	
Sex^b^	Male	36/82 (43.9)	1.0	0.842	1.0	0.850
	Female	45/106 (42.5)	0.94 (0.53–1.69)		0.94 (0.52–1.72)	
Education level^c^	Complete secondary or higher	70/172 (40.7)	1.0	0.030	1.0	0.040
	Incomplete secondary or lower	11/16 (68.8)	3.21 (1.07–9.63)		3.17 (1.01–9.96)	
Role in HCF^d^	Clinician, nurse, midwife	68/156 (43.6)	1.0	0.431	1.0	0.327
	Clinical support staff	7/13 (53.9)	1.51 (0.49–4.70)		1.85 (0.57–6.00)	
	Other staff	6/19 (31.6)	0.60 (0.22–1.65)		0.59 (0.21–1.70)	
Perceptions of the impact of the COVID-19 pandemic and the COVID-19 response in Goma ^e^						
Healthcare services are suffering^f^	No/don’t know	52/149 (34.9)	1.0	<0.001	1.0	<0.001
	Yes	29/39 (74.4)	5.41 (2.45–11.96)		5.69 (2.45–13.21)	
The response is causing neglect of other diseases^f^	No/don’t know	55/156 (35.3)	1.0	<0.001	1.0	<0.001
	Yes	26/32 (81.3)	7.96 (3.09–20.50)		8.61 (3.24–22.89)	
The response is detrimental to the economy^f^	No/don’t know	37/109 (33.9)	1.0	0.003	1.0	0.010
	Yes	44/79 (55.7)	2.45 (1.35–4.44)		2.24 (1.21–4.17)	
The response is detrimental to education^f^	No/don’t know	20/48 (41.7)	1.0	0.818	1.0	0.980
	Yes	61/140 (43.6)	1.08 (0.56–2.10)		1.01 (0.50–2.02)	
Understanding and views of COVID-19 vaccines						
The vaccines protect against SARS-CoV-2 infection^f^	No	52/146 (35.6)	1.0	<0.001	1.0	<0.001
	Yes	29/42 (69.1)	4.03 (1.93–8.42)		3.69 (1.73–7.87)	
The vaccines stop people getting very ill from COVID-19^f^	No	52/146 (35.6)	1.0	<0.001	1.0	<0.001
	Yes	29/42 (69.1)	4.03 (1.93–8.42)		3.69 (1.73–7.87)	
The vaccines are important for control of the pandemic^f^	No/don’t know	3/47 (6.4)	1.0	<0.001	1.0	<0.001
	Yes	78/141 (55.3)	18.16 (5.38–61.25)		18.19 (5.23–63.30)	
The vaccines are new and experimental^f^	No	7/22 (31.8)	1.0	0.250	1.0	0.172
	Yes	74/166 (44.6)	1.72 (0.67–4.45)		1.98 (0.72–5.41)	
The vaccines may not work^f^	No/don’t know	60/146 (41.1)	1.0	0.306	1.0	0.551
	Yes	21/42 (50.0)	1.43 (0.72–2.85)		1.24 (0.61–2.54)	
The vaccines may cause side effects or other harm^f^	No	25/59 (42.4)	1.0	0.894	1.0	0.896
	Yes	56/129 (43.4)	1.04 (0.56–1.94)		1.04 (0.55–2.00)	

N, number; OR, odds ratio; CI, confidence interval; LRT, likelihood ratio test; HCF, healthcare facility.

a Adjusted for sex, education level and role in the facility.

b Adjusted for age category, education level and role in the facility.

c Adjusted for age category, sex and role in the facility.

d Adjusted for age category, sex and education level.

e Where the “COVID-19 response” refers to the country level measures that were put in place in order to control the spread of SARS-CoV-2 and respond to the burden of COVID-19 disease.

f Adjusted for age category, sex, education level and role in the healthcare facility.

Whilst many participants from Goma had concerns that COVID-19 vaccines were new and experimental (*n* = 166; 88.3%), that they might not work (*n* = 42; 22.3%), and that they may cause side effects or harm (*n* = 129; 68.6%), these concerns were not associated with lower intention to get vaccinated in multivariable analyses (Table 5). Thus, whilst these concerns may have played an integral role in decision making among participants who did not intend to get vaccinated, the concerns were similarly prevalent among participants who reported intent to get vaccinated (i.e., with the latter group intending to get vaccinated despite these concerns).

Additional exploratory analyses evaluated factors associated with uptake of ≥1 vaccine dose in Masaka, with the hypothesis that unvaccinated participants may be those who had delayed getting vaccinated (as the survey was conducted 6–7 months after vaccine introduction); none of the variables evaluated were associated with uptake (Supplementary Table S4).

## Discussion

To our knowledge, this was the first study in SSA to evaluate acceptability of COVID-19 vaccines (alongside participants’ knowledge and perceptions of, and attitudes towards, the vaccines) in a diverse range of healthcare facility workers with varying levels of patient contact, and to stratify findings across cadres. Consistent with earlier studies from SSA [16], reported COVID-19 vaccine acceptability was high in Kambia and Masaka, with most participants having already received ≥1 vaccine dose or reporting that they would have the vaccine if offered it.

Accounting for the timing of the survey, COVID-19 vaccine uptake (of ≥1 dose) was greatest in Kambia (where the survey was conducted 1–3 months after vaccine introduction), possibly reflecting high country-wide willingness to get vaccinated following the devastation of the 2014–2015 West African Ebola outbreak. In 2015, research infrastructure was rapidly set up in Kambia for conduct of clinical trials of novel Ebola vaccines [18, 19]; a programme of these trials continues to date. Thus, the high COVID-19 vaccine acceptability observed among healthcare facility workers in Kambia may additionally be motivated by 7 years of research teams’ engagement, communication and building trust with and between local authorities, communities and healthcare personnel [20, 21]. Whilst concerns about adverse effects of COVID-19 vaccines were common across all 3 study sites, they were most common in Kambia. Thus, the high vaccine acceptability observed despite these concerns suggests a high level of trust in authorities, vaccine manufacturers and other relevant institutions.

It is notable that many participants from Masaka were reportedly willing to have a COVID-19 vaccine but not yet vaccinated at the time of the survey (conducted 6–7 months after vaccine introduction) given that healthcare workers were prioritized for vaccination. For comparison, a study conducted in Malawi between 0.5 and 2 months after COVID-19 vaccine introduction in the country found that 83% of surveyed healthcare workers had already received the first COVID-19 vaccine dose [22]. Whilst the slower uptake in Masaka may reflect logistical constraints and delays in vaccine roll-out and challenges in vaccine access, it may also be due to participants delaying vaccination or decision-making on whether to get vaccinated. In Masaka, the main COVID-19 vaccine available to participants at the time of the survey was the Covishield AstraZeneca vaccine (produced by the Serum Institute of India), and there was extensive media attention surrounding a small risk of thrombotic events associated with the AstraZeneca COVID-19 vaccine around that time [23, 24]. Furthermore, there were long and heavily publicised delays in the European Medicines Agency (EMA) issuing approval of the Covishield vaccine, with ensuing challenges to entering Europe for travellers who had received it [25]. Anecdotally, many people in Uganda reported waiting for “better” or “safer” COVID-19 vaccines to become available before getting vaccinated. Nonetheless, our results are consistent with earlier research from Uganda that showed high acceptability of COVID-19 vaccines in general among eye healthcare workers [26] and high willingness of healthcare workers to participate in COVID-19 vaccine trials [27].

COVID-19 vaccine introduction in the DRC met with several challenges. The vaccine launch was initially scheduled for March 2021 but postponed following the temporary suspensions in roll-out of the AstraZeneca vaccine in Europe (due to the small risk of thrombotic events described above) [24]. Whilst the vaccination campaign in the DRC started just a month later, rapid and extensive spread of vaccine-related rumours across the country and the president’s vociferous mistrust in the vaccines meant that uptake was very slow [28, 29]. This is reflected in the results of this study, with no study participants from Goma having received a COVID-19 vaccine by the time of the survey (conducted 2–3 months after vaccinations started), and less than half reporting willingness to be vaccinated. A public health official from the DRC expressed concerns that distrust in the vaccines was amplified by the “unfortunate communication around AstraZeneca in Europe” [30]. Nonetheless, the poor uptake of COVID-19 vaccines in the DRC follows a history of multiple outbreaks of measles and yellow fever occurring in the country over the past decade due to inadequate coverage of the respective vaccines, partly resulting from low vaccine acceptability [28].

In earlier studies, female gender, lower age and being in a nursing role were associated with COVID-19 vaccine hesitancy among healthcare workers, and being older, male and a doctor were associated with higher vaccine acceptability [31–34]. These associations were not observed in this study. However, reported intent to get vaccinated in Goma was associated with perceptions that the COVID-19 pandemic and the country’s response to the pandemic were having negative impacts on the country (for example, on healthcare services or the economy) and, as seen in previous studies of healthcare workers in Malawi, South Africa and Ethiopia [22, 34, 35], with positive perceptions of the vaccines’ efficacy/effectiveness and impact against SARS-CoV-2 infection or COVID-19 disease.

A major strength of this study is that it was conducted in sites from 3 countries spanning East, Central and West Africa, and that it included participants from a range of healthcare facilities in each of the study locations. Furthermore, the study included a wide variety of healthcare facility workers with patient contact roles and results were stratified by cadre. The survey was conducted at an important time within the context of vaccine roll-out in SSA; the vaccines were newly available in the countries and, given that healthcare workers were prioritized for vaccination, most participants should have had access to the vaccine or would be given access within the near future. Several earlier studies of healthcare workers were conducted before the COVID-19 vaccines became available in their country [15], and thus reported acceptability was hypothetical.

The cross-sectional study design was a limitation as results represent participant views at just one point in time. Vaccine decision-making is a dynamic process and vaccination status or intent to get vaccinated may change over time. This is particularly relevant for a newly available vaccine as many people may be reassured as more populations are vaccinated and as more safety/efficacy data become available. Second, it was not always possible to verify vaccination status using vaccination cards. The risk of recall bias with self-reporting of vaccination status is likely to be low in this study, but social desirability bias is possible. Finally, due to the quantitative study design, it was not possible to explore the in-depth nuances of participants’ views and perceptions and rationale behind their decision-making. There were also several inconsistencies in participants’ responses that could not be further explored and elucidated. Nonetheless, the study highlights interesting and important topics that could be further explored through employment of qualitative methods.

### Conclusion

In 2019, the WHO declared vaccine hesitancy as one of the ten greatest threats to global health [36]. With the recent development, trialling and roll-out of COVID-19 vaccines, topics surrounding vaccine decision-making, misinformation and ‘anti-vax’ movements have gained even greater prominence in both the scientific literature and the media. This survey provides important results on COVID-19 vaccine acceptability among healthcare facility workers in the months following vaccine introduction in study sites from 3 SSA countries, which may be informative for future vaccine introductions in the region, particularly within the context of an outbreak or pandemic. The results demonstrate the importance of educating healthcare staff on vaccine development processes, and on evidence surrounding vaccine efficacy/effectiveness and safety, both to promote vaccine uptake in this group and to ensure appropriate and accurate communication regarding vaccines between healthcare staff and their patients or the wider communities. Results also demonstrate the importance of careful and accurate messaging surrounding vaccines and vaccine introductions at a global level.
