# Supplementary material for: COVID-19 Vaccine Acceptability Among Healthcare Facility Workers in Sierra Leone, the Democratic Republic of Congo and Uganda: A Multi-Centre Cross-Sectional Survey
Source: Int J Public Health. 2022 Sep 23;67:1605113. doi: 10.3389/ijph.2022.1605113 (PMC9537362; doi:10.3389/ijph.2022.1605113)
Supplement: Supplementary file 1 [file DataSheet1.docx]

**SUPPLEMENTARY MATERIAL**

**Table S1: Questionnaire data by role within healthcare facility in Kambia.** (COVID-19 vaccination in health workers; Sierra Leone, Democratic Republic of Congo and Uganda; 2021)

|  | **Clinicians, nurses & midwives,** N=29 | **Clinical support staff,** N=71 | **Other staff**  N=24 |
| --- | --- | --- | --- |
| **Age in years**, median (range) | 43 (27-59) | 37 (21-58) | 36 (20-68) |
| **Gender**  Male  Female | 2 (6.9)  27 (93.1) | 25 (35.2)  46 (64.8) | 17 (70.8)  7 (29.2) |
| **Highest level of schooling**  None  Complete primary  Incomplete secondary  Complete secondary & above | 0  0  2 (6.9)  27 (93.1) | 1 (1.4)  0  22 (31.0)  48 (67.6) | 6 (25.0)  1 (4.2)  7 (29.2)  10 (41.7) |
| **Perceptions of COVID-19 as a public health problem in Sierra Leone** ^a^  COVID-19 is an important public health problem  Many people are getting sick from COVID-19  Many people are dying from COVID-19  Many HCW are getting sick from COVID-19  Many HCW are dying from COVID-19  HCF are overwhelmed with COVID-19 cases | 29 (100.0)  26 (89.7)  16 (55.2)  24 (82.8)  16 (55.2)  15 (51.7) | 71 (100.0)  69 (97.2)  43 (60.6)  65 (91.6)  44 (62.0)  29 (40.9) | 24 (100.0)  22 (91.7)  16 (66.7)  21 (87.5)  15 (62.5)  13 (54.2) |
| **Perceptions of COVID-19 impact in Sierra Leone** ^a^  Healthcare services are suffering  Other diseases are more important  COVID-19 response is causing neglect of other diseases  COVID-19 response is detrimental to the economy  COVID-19 response is detrimental to education | 13 (44.8)  22 (75.9)  13 (44.8)  27 (93.1)  15 (51.7) | 35 (49.3)  38 (53.5)  30 (42.3)  70 (98.6)  36 (50.7) | 17 (70.8)  18 (75.0)  11 (45.8)  22 (91.7)  14 (58.3) |
| **Knowledge and perceptions of COVID-19 vaccines** ^a^  They are under development / being evaluated  They are licensed and used in some countries  They are available in this country  They protect against virus that causes COVID-19  They stop people getting very sick from COVID-19  They are important for control of the pandemic | 27 (93.1)  29 (100.0)  21 (72.4)  29 (100.0)  29 (100.0)  29 (100.0) | 68 (95.8)  69 (97.2)  47 (66.2)  66 (93.0)  64 (90.1)  71 (100.0) | 22 (91.7)  24 (100.0)  18 (75.0)  21 (87.5)  23 (95.8)  24 (100.0) |
| **Advantages of COVID-19 vaccines** ^a^  They can/may help to control Covid-19  They can/may protect healthcare workers  They can be given to lots of people quickly  They may allow travel/movement/socializing  They may encourage visitors from other countries  They may allow the economy to recover | 29 (100.0)  28 (96.6)  28 (96.6)  29 (100.0)  29 (100.0)  18 (62.1) | 71 (100.0)  71 (100.0)  65 (91.6)  71 (100.0)  70 (98.6)  54 (76.1) | 24 (100.0)  24 (100.0)  24 (100.0)  23 (95.8)  24 (100.0)  20 (83.3) |
| **Disadvantages of COVID-19 vaccines** ^a^  Vaccine rollout is a burden on healthcare services  They are new and experimental  They may cause side effects/harm  Multidose vaccine schedules are difficult to deliver  They are expensive; big cost to the country | 27 (93.1)  28 (96.6)  29 (100.0)  28 (96.6)  28 (96.6) | 61 (85.9)  62 (87.3)  71 (100.0)  62 (87.3)  63 (88.7) | 23 (95.8)  23 (95.8)  24 (100.0)  23 (95.8)  21 (87.5) |
| **Concerns over COVID-19 vaccines** ^a^  They may cause infertility  They may cause SARS-CoV-2 infection/COVID-19  They may cause symptoms like COVID-19  They may affect pregnancies/foetuses  They may cause anaphylaxis  They may cause other harm  They may not work | 26 (89.7)  26 (89.7)  26 (89.7)  26 (89.7)  26 (89.7)  25 (86.7)  24 (82.7) | 49 (69.0)  48 (67.6)  48 (67.6)  50 (70.4)  50 (70.4)  49 (69.0)  47 (66.2) | 20(83.3)  17 (70.8)  19 (79.2)  20 (83.3)  20 (83.3)  17 (70.8)  18 (75.0) |
| **COVID-19 vaccination status**  Fully vaccinated ^b^  Partially vaccinated ^b^  Unvaccinated – would accept vaccine  Unvaccinated – would not accept vaccine | 4 (13.8)  13 (44.8)  11 (37.9)  1 (3.5) ^d^ | 3 (4.2)  22 (31.0)  46 (64.8)  0 | 2 (8.3)  4 (16.7)  18 (75.0)  0 |
| **Reasons for accepting or being willing to accept the vaccine** ^a^**^,^**^c^  To protect own health  To keep working  To avoid infecting other people  To protect family | 28 (96.6)  28 (96.6)  28 (96.6)  28 (96.6) | 71 (100.0)  70 (98.6)  71 (100.0)  70 (98.6) | 24 (100.0)  24 (100.0)  24 (100.0)  24 (100.0) |
| **Factors that would influence decision on whether to get vaccinated** ^a,e^  Which country the vaccine was developed in  Which company made the vaccine  Where the clinical trials were conducted  Whether clinical trials were conducted here  How many people were vaccinated before  How long the vaccine has been trialled for  What type of vaccine it is  Which other countries are giving the vaccine | 23 (79.3)  17 (58.6)  22 (75.9)  19 (65.5)  18 (62.1)  25 (86.2)  18 (62.1)  12 (41.4) | 57 (80.3)  56 (78.9)  58 (81.7)  55 (77.5)  55 (77.5)  64 (90.1)  51 (71.8)  34 (47.9) | 20 (83.3)  19 (79.2)  18 (75.0)  18 (75.0)  18 (75.0)  21 (87.5)  16 (66.7)  9 (37.5) |

N: Number; HCF: Healthcare facility; HCW: Healthcare workers.

^a^ Participants could provide more than one answer so summed percentages are not equal to 100%.

^b^ In Kambia, vaccination status was confirmed using vaccination cards.

^c^ Participants provided reasons for or against vaccination as appropriate to their answer for the vaccination status variable above, but proportions were calculated as percentages of the whole study population (by role).

^d^ The participant who was unwilling to be vaccinated felt that they were not at risk of catching SARS-CoV-2, they were not at risk of dying or becoming ill from COVID-19, and they were frightened of vaccine side effects.

^e^ Results are shown for all participants, including participants who had already received one or more COVID-19 vaccine doses.

**Table S2: Questionnaire data by role within healthcare facility in Goma.** (COVID-19 vaccination in health workers; Sierra Leone, Democratic Republic of Congo and Uganda; 2021)

|  | **Clinicians, nurses & midwives,** N=156 | **Clinical support staff,** N=13 | **Other staff**  N=19 |
| --- | --- | --- | --- |
| **Age in years**, median (range) | 40 (22-75) | 28 (22-52) | 33 (21-52) |
| **Gender**  Male  Female | 69 (44.2)  87 (55.8) | 5 (38.5)  8 (61.5) | 8 (42.1)  11 (57.9) |
| **Highest level of schooling**  Complete primary  Incomplete secondary  Complete secondary & above | 1 (0.6)  12 (7.7)  143 (91.7) | 0  1 (7.7)  12 (92.3) | 0  2 (10.5)  17 (89.5) |
| **Perceptions of COVID-19 as a public health problem in the DRC** ^a^  COVID-19 is an important public health problem  Many people are getting sick from COVID-19  Many people are dying from COVID-19  Many HCW are getting sick from COVID-19  Many HCW are dying from COVID-19  HCF are overwhelmed with COVID-19 cases | 153 (98.1)  134 (85.9)  119 (76.3)  69 (44.2)  45 (28.9)  23 (14.7) | 13 (100.0)  12 (92.3)  12 (92.3)  5 (38.5)  2 (15.4)  2 (15.4) | 19 (100.0)  15 (79.0)  12 (63.2)  7 (36.8)  3 (15.8)  1 (5.3) |
| **Perceptions of COVID-19 impact in the DRC** ^a^  Healthcare services are suffering  Other diseases are more important  COVID-19 response is causing neglect of other diseases  COVID-19 response is detrimental to the economy  COVID-19 response is detrimental to education | 33 (21.2)  70 (44.9)  27 (17.3)  66 (42.3)  119 (76.3) | 1 (7.7)  4 (30.8)  1 (7.7)  5 (38.5)  7 (53.9) | 5 (26.3)  7 (36.8)  4 (21.1)  8 (42.1)  14 (73.7) |
| **Knowledge and perceptions of COVID-19 vaccines** ^a^  They are under development / being evaluated  They are licensed and used in some countries  They are available in this country  They protect against virus that causes COVID-19  They stop people getting very sick from COVID-19  They are important for control of the pandemic | 137 (87.8)  100 (64.1)  121 (77.6)  36 (23.1)  36 (23.1)  118 (75.6) | 12 (92.3)  9 (69.2)  10 (76.9)  2 (15.4)  2 (15.4)  9 (69.2) | 16 (84.2)  11 (57.9)  13 (68.4)  4 (21.1)  4 (21.1)  14 (73.7) |
| **Advantages of COVID-19 vaccines** ^a^  They can/may help to control COVID-19  They can/may protect healthcare workers  They can be given to lots of people quickly  They may allow travel/movement/socializing  They may encourage visitors from other countries  They may allow the economy to recover | 111 (71.2)  120 (76.9)  34 (21.8)  104 (66.7)  96 (61.5)  73 (46.8) | 9 (69.2)  10 (76.9)  5 (38.5)  8 (61.5)  7 (53.9)  7 (53.9) | 14 (73.7)  17 (89.5)  4 (21.1)  16 (84.2)  15 (79.0)  9 (47.4) |
| **Disadvantages of COVID-19 vaccines** ^a^  Vaccine rollout is a burden on healthcare services  They are new and experimental  They may cause side effects/harm  Multidose vaccine schedules are difficult to deliver  They are expensive; big cost to the country | 34 (21.8)  137 (87.8)  108 (69.2)  21 (13.5)  38 (24.4) | 4 (30.8)  12 (92.3)  7 (53.9)  1 (7.7)  5 (38.5) | 6 (31.6)  17 (89.5)  14 (73.7)  2 (10.5)  7 (36.8) |
| **Concerns over COVID-19 vaccines** ^a^  They may cause infertility  They may cause SARS-CoV-2 infection/COVID-19  They may cause symptoms like COVID-19  They may affect pregnancies/foetuses  They may cause anaphylaxis  They may cause other harm  They may not work | 31 (19.9)  86 (55.1)  105 (67.3)  78 (50.0)  103 (66.0)  86 (55.1)  36 (23.1) | 3 (23.1)  6 (46.2)  10 (76.9)  8 (61.5)  7 (53.9)  7 (53.9)  3 (23.1) | 5 (26.3)  15 (79.0)  17 (89.5)  13 (68.4)  10 (52.6)  12 (63.2)  3 (15.8) |
| **COVID-19 vaccination status**  Unvaccinated – would accept vaccine  Unvaccinated – would not accept vaccine | 68 (43.6)  88 (56.4) | 7 (53.9)  6 (46.2) | 6 (31.6)  13 (68.4) |
| **Reasons for accepting or being willing to accept the vaccine** ^a,b^  To protect own health  To keep working  To avoid infecting other people  To protect family | 68 (43.6)  38 (24.4)  66 (42.3)  63 (40.4) | 7 (53.9)  5 (38.5)  7 (53.9)  7 (53.9) | 6 (31.6)  6 (31.6)  6 (31.6)  6 (31.6) |
| **Reasons for unwillingness to accept vaccine** ^a,b^  Not at risk of catching SARS-CoV-2  Not at risk of becoming very ill or dying from COVID-19  Already had COVID-19  Frightened of vaccine side effects  COVID-19 vaccines are new/experimental  COVID-19 vaccines don’t work  Currently pregnant/breastfeeding | 15 (9.6)  14 (9.0)  2 (1.3)  61 (39.1)  61 (39.1)  25 (16.0)  3 (1.9) | 2 (15.4)  2 (15.4)  0  4 (30.8)  3 (23.1)  1 (7.7)  0 | 1 (5.3)  2 (10.5)  0  9 (47.4)  11 (57.9)  4 (21.1)  0 |
| **Factors that would influence decision on whether to get vaccinated** ^a,c^  Which country the vaccine was developed in  Which company made the vaccine  Where the clinical trials were conducted  Whether clinical trials were conducted here  How many people were vaccinated before  How long the vaccine has been trialled for  What type of vaccine it is  Which other countries are giving the vaccine | 87 (55.8)  71 (45.5)  112 (71.8)  93 (59.6)  103 (66.0)  109 (69.9)  62 (39.7)  70 (44.9) | 10 (76.9)  10 (76.9)  13 (100.0)  12 (92.3)  11 (84.6)  12 (92.3)  4 (30.8)  10 (76.9) | 8 (42.1)  7 (36.8)  13 (68.4)  13 (68.4)  13 (68.4)  13 (68.4)  7 (37.8)  10 (52.6) |

N: Number; HCF: Healthcare facility; HCW: Healthcare workers.

^a^ Participants could provide more than one answer so summed percentages are not equal to 100%.

^b^ Participants provided either reasons for or against vaccination as appropriate to their answer for the vaccination status variable above, but proportions were calculated as percentages of the whole study population (by role).

^c^ Results are shown for all participants, including participants who had already received one or more COVID-19 vaccine doses.

**Table S3: Questionnaire data by role within healthcare facility in Masaka.** (COVID-19 vaccination in health workers; Sierra Leone, Democratic Republic of Congo and Uganda; 2021)

|  | **Clinicians, nurses & midwives,** N=132 | **Clinical support staff,** N=48 | **Other staff**  N=51 |
| --- | --- | --- | --- |
| **Age in years**, median (range) | 36 (18-74) | 35 (23-67) | 35 (22-61) |
| **Gender**  Male  Female | 23 (17.4)  109 (82.6) | 19 (39.6)  29 (60.4) | 31 (60.8)  20 (39.2) |
| **Highest level of schooling**  None  Complete primary  Incomplete secondary  Complete secondary & above | 0  0  6 (4.6)  126 (95.5) | 0  5 (10.4)  12 (25.0)  31 (64.6) | 1 (2.0)  2 (3.9)  2 (3.9)  46 (90.2) |
| **Perceptions of COVID-19 as a public health problem in Uganda** ^a^  COVID-19 is an important public health problem  Many people are getting sick from COVID-19  Many people are dying from COVID-19  Many HCW are getting sick from COVID-19  Many HCW are dying from COVID-19  HCF are overwhelmed with COVID-19 cases | 129 (97.7)  101 (76.5)  80 (60.6)  80 (60.6)  57 (43.2)  58 (43.9) | 45 (93.8)  35 (72.9)  34 (70.8)  32 (66.7)  17 (35.4)  21 (43.8) | 51 (100.0)  41 (80.4)  33 (64.7)  34 (66.7)  29 (56.9)  28 (54.9) |
| **Perceptions of COVID-19 impact in Uganda** ^a^  Healthcare services are suffering  Other diseases are more important  COVID-19 response is causing neglect of other diseases  COVID-19 response is detrimental to the economy  COVID-19 response is detrimental to education | 99 (75.0)  21 (15.9)  80 (60.6)  116 (87.9)  125 (94.7) | 42 (87.5)  15 (31.3)  26 (54.2)  41 (85.4)  44 (91.7) | 43 (84.3)  9 (17.6)  29 (56.9)  42 (82.4)  47 (92.2) |
| **Knowledge and perceptions of COVID-19 vaccines** ^a^  They are under development / being evaluated  They are licensed and used in some countries  They are available in this country  They protect against virus that causes Covid-19  They stop people getting very sick from Covid-19  They are important for control of the pandemic | 60 (45.5)  89 (67.4)  105 (79.5)  62 (47.0)  71 (53.8)  127 (96.2) | 25 (52.1)  26 (54.2)  31 (64.6)  24 (50.0)  25 (52.1)  45 (93.8) | 31 (60.8)  38 (74.5)  40 (78.4)  34 (66.7)  27 (52.9)  50 (98.0) |
| **Advantages of COVID-19 vaccines** ^a^  They can/may help to control Covid-19  They can/may protect healthcare workers  They can be given to lots of people quickly  They may allow travel/movement/socializing  They may encourage visitors from other countries  They may allow the economy to recover | 117 (88.6)  111 (84.1)  47 (35.6)  90 (68.2)  78 (59.1)  84 (63.6) | 41 (85.4)  37 (77.1)  18 (37.5)  28 (58.3)  31 (64.6)  23 (47.9) | 45 (88.2)  45 (88.2)  29 (56.9)  41 (80.4)  38 (74.5)  31 (60.8) |
| **Disadvantages of COVID-19 vaccines** ^a^  Vaccine rollout is a burden on healthcare services  They are new and experimental  They may cause side effects/harm  Multidose vaccine schedules are difficult to deliver  They are expensive; big cost to the country | 35 (26.5)  58 (43.9)  82 (62.1)  48 (36.4)  75 (56.8) | 10 (20.8)  22 (45.8)  30 (62.5)  17 (35.4)  18 (37.5) | 17 (33.3)  34 (66.7)  37 (72.6)  19 (37.3)  32 (62.8) |
| **Concerns over COVID-19 vaccines** ^a^  They may cause infertility  They may cause SARS-CoV-2 infection/COVID-19  They may cause symptoms like COVID-19  They may affect pregnancies/foetuses  They may cause anaphylaxis  They may cause other harm  They may not work | 23 (17.4)  35 (26.5)  72 (54.6)  56 (42.4)  45 (34.1)  27 (20.5)  20 (15.2) | 3 (6.3)  7 (14.6)  17 (35.4)  16 (33.3)  11 (22.9)  6 (12.5)  6 (12.5) | 7 (13.7)  10 (19.6)  22 (43.1)  16 (31.4)  12 (23.5)  9 (17.7)  6 (11.8) |
| **COVID-19 vaccination status**  Fully vaccinated ^b^  Partially vaccinated ^b^  Unvaccinated – would accept vaccine  Unvaccinated – would not accept vaccine | 17 (12.9)  33 (25.0)  80 (60.6)  2 (1.5) | 8 (16.7)  13 (27.1)  26 (54.2)  1 (2.1) | 9 (17.7)  9 (17.7)  30 (58.8)  3 (5.9) |
| **Reasons for accepting or being willing to accept the vaccine** ^a,c^  To protect own health  To keep working  To avoid infecting other people  To protect family | 121 (91.7)  59 (44.7)  92 (69.7)  102 (77.3) | 46 (95.8)  20 (41.7)  29 (60.4)  33 (68.8) | 47 (92.2)  21 (41.2)  40 (78.4)  37 (72.6) |
| **Reasons for unwillingness to accept vaccine** ^a,c^  Not at risk of catching SARS-CoV-2  Not at risk of becoming very ill or dying from COVID-19  Already had COVID-19  Frightened of vaccine side effects  COVID-19 vaccines are new/experimental  COVID-19 vaccines don’t work  Currently pregnant/breastfeeding | 1 (0.8)  0  0  2 (1.5)  3 (2.3)  2 (1.5)  0 | 0  0  0  1 (2.1)  1 (2.1)  0  0 | 1 (2.0)  0  1 (2.0)  2 (3.9)  2 (3.9)  0  0 |
| **Factors that would influence decision on whether to get vaccinated** ^a,d^  Which country the vaccine was developed in  Which company made the vaccine  Where the clinical trials were conducted  Whether clinical trials were conducted here  How many people were vaccinated before  How long the vaccine has been trialled for  What type of vaccine it is  Which other countries are giving the vaccine | 79 (59.9)  58 (43.9)  53 (40.2)  44 (33.3)  42 (31.8)  57 (43.2)  62 (47.0)  38 (28.8) | 17 (35.4)  14 (29.2)  23 (47.9)  15 (31.3)  13 (27.1)  32 (66.7)  22 (45.8)  15 (31.3) | 33 (64.7)  28 (54.9)  29 (56.9)  28 (54.9)  18 (35.3)  22 (43.1)  25 (49.0)  19 (37.3) |

N: Number; HCF: Healthcare facility; HCW: Healthcare workers.

^a^ Participants could provide more than one answer so summed percentages are not equal to 100%.

^b^ In Masaka, vaccination status was confirmed using vaccination cards or verbally.

^c^ Participants provided either reasons for or against vaccination as appropriate to their answer for the vaccination status variable above, but proportions were calculated as percentages of the whole study population (by role).

^d^ Results are shown for all participants, including participants who had already received one or more COVID-19 vaccine doses.

**Table S4: Factors associated with COVID-19 vaccine uptake in Masaka.** (COVID-19 vaccination in health workers; Sierra Leone, Democratic Republic of Congo and Uganda; 2021)

| **Variable** | **Category** | **N vaccinated / Total (%)** | **Crude OR**  **(95% CI)** | **LRT**  **p-value** | **Adjusted OR**  **(95% CI)** | **LRT**  **p-value** |
| --- | --- | --- | --- | --- | --- | --- |
| **Age in years** ^a^ | ≤35  36-50  >50 | 45 / 115 (39.1)  30 / 85 (35.3)  14 / 30 (46.7) | 1.0  0.85 (0.47-1.52)  1.36 (0.61-3.06) | 0.545 | 1.0  0.72 (0.39-1.34)  0.97 (0.39-2.42) | 0.547 |
| **Sex** ^b^ | Male  Female | 25 / 73 (34.3)  64 / 158 (40.5) | 1.0  1.31 (0.73-2.33) | 0.361 | 1.0  1.45 (0.77-2.73) | 0.253 |
| **Education level** ^c^ | Complete secondary or higher  Incomplete secondary or lower | 74 / 203 (36.5)  15 / 28 (53.6) | 1.0  2.01 (0.91-4.46) | 0.085 | 1.0  2.21 (0.84-5.80) | 0.105 |
| **Role in HCF** ^d^ | Clinician, nurse, midwife  Clinical support staff  Other staff | 50 / 132 (37.9)  21 / 48 (43.8)  18 / 51 (35.3) | 1.0  1.28 (0.65-2.49)  0.89 (0.46-1.75) | 0.672 | 1.0  1.02 (0.48-2.18)  0.99 (0.47-2.10) | 0.998 |
| **Perceptions of the impact of the COVID-19 pandemic and the COVID-19 response in Masaka** | | | | | | |
| **Healthcare services are suffering** ^e^ | No / don’t know  Yes | 18 / 47 (38.3)  71 / 184 (38.6) | 1.0  1.01 (0.52-1.96) | 0.971 | 1.0  0.96 (0.49-1.89) | 0.914 |
| **The response is causing neglect of other diseases** ^e^ | No / don’t know  Yes | 41 / 96 (42.7)  48 / 135 (35.6) | 1.0  0.75 (0.50-1.12) | 0.272 | 1.0  0.70 (0.40-1.21) | 0.204 |
| **The response is detrimental to the economy** ^e^ | No / don’t know  Yes | 12 / 32 (37.5)  77 / 199 (38.7) | 1.0  1.05 (0.49-2.27) | 0.897 | 1.0  1.00 (0.45-2.20) | 0.999 |
| **The response is detrimental to education** ^e,f^ | No / don’t know  Yes | 5 / 15 (33.3)  84 / 216 (38.9) | 0.79 (0.26-2.38)  1.0 | 0.666 | 0.84 (0.27-2.60)  1.0 | 0.759 |
| **Understanding and views of COVID-19 vaccines** | | | | | | |
| **The vaccines protect against SARS-CoV-2 infection** ^e^ | No  Yes | 37 111 (33.3)  52 / 120 (43.3) | 1.0  1.53 (0.90-2.61) | 0.118 | 1.0  1.59 (0.92-2.76) | 0.097 |
| **The vaccines stop people getting very ill from COVID-19** ^e^ | No  Yes | 43 / 108 (39.8)  46 / 123 (37.4) | 1.0  0.90 (0.53-1.54) | 0.71 | 1.0  0.92 (0.54-1.59) | 0.773 |
| **The vaccines are important for control of the pandemic** ^e,f^ | No / don’t know  Yes | 2 / 9 (22.2)  87 / 222 (39.2) | 0.44 (0.09-2.18)  1.0 | 0.287 | 0.40 (0.08-2.03)  1.0 | 0.238 |
| **The vaccines are new and experimental** ^e^ | No  Yes | 43 / 117 (36.8)  46 / 114 (40.4) | 1.0  1.16 (0.69-1.98) | 0.574 | 1.0  1.33 (0.76-2.34) | 0.312 |
| **The vaccines may not work** ^e^ | No / don’t know  Yes | 77 / 199 (38.7)  12 / 32 (37.5) | 1.0  0.95 (0.44-2.05) | 0.897 | 1.0  0.96 (0.44-1.12) | 0.923 |
| **The vaccines may cause side effects or other harm** ^e^ | No  Yes | 30 / 82 (36.6)  59 / 149 (39.6) | 1.0  1.14 (0.65-1.98) | 0.652 | 1.0  1.21 (0.67-2.20) | 0.524 |

N: Number; OR: Odds ratio; CI: Confidence interval; LRT: Likelihood ratio test; HCF: Healthcare facility;.

^a^ Adjusted for sex, education level and role in the facility.

^b^ Adjusted for age category, education level and role in the facility.

^c^ Adjusted for age category, sex and role in the facility.

^d^ Adjusted for age category, sex and education level.

^e^ Adjusted for age category, sex, education level and role in the healthcare facility.

^f^ The ‘yes’ category was used as the reference category for these variables due to data sparsity in the ‘No / don’t know’ category.

**Figure S1: Reported vaccine acceptability by country of vaccine development among participants from Kambia (black bars), Goma (striped bars) and Masaka (white bars).** Bars show the proportion of participants per country who reported that they would theoretically accept a COVID-19 vaccine that was developed in the United States of America (USA), the United Kingdom (UK), Europe, China or Russia. (COVID-19 vaccination in health workers; Sierra Leone, Democratic Republic of Congo and Uganda; 2021)
